# Seven in Absentia E3 Ubiquitin Ligases: Central Regulators of Neural Cell Fate and Neuronal Polarity

**DOI:** 10.3389/fncel.2017.00322

**Published:** 2017-10-13

**Authors:** Taren Ong, David J. Solecki

**Affiliations:** ^1^Cancer and Developmental Biology Track, Integrated Biomedical Sciences Graduate Program, University of Tennessee Health Science Center, Memphis, TN, United States; ^2^Department of Developmental Neurobiology, St. Jude Children’s Research Hospital, Memphis, TN, United States

**Keywords:** ubiquitin ligase, seven in absentia, neuronal polarity, cerebellar granule neuron, Pard complex

## Abstract

During neural development, neural precursors transition from a proliferative state within their germinal niches to a migratory state as they relocate to their final laminar positions. Transitions across these states are coupled with dynamic alterations in cellular polarity. This key feature can be seen throughout the developing vertebrate brain, in which neural stem cells give rise to multipolar or unpolarized transit-amplifying progenitors. These transit-amplifying progenitors then expand to give rise to mature neuronal lineages that become polarized as they initiate radial migration to their final laminar positions. The conventional understanding of the cellular polarity regulatory program has revolved around signaling cascades and transcriptional networks. In this review, we discuss recent discoveries concerning the role of the Siah2 ubiquitin ligase in initiating neuronal polarity during cerebellar development. Given the unique features of Siah ubiquitin ligases, we highlight some of the key substrates that play important roles in cellular polarity and propose a function for the Siah ubiquitin proteasome pathway in mediating a post-translational regulatory network to control the onset of polarization.

## Introduction

A salient feature of neurogenesis is the expansion of neural precursors within their germinal niches. This expansion is followed by cell cycle exit, cellular differentiation, and migration of the cells to their distant sites of function (Hatten, 1999). In the vertebrate brain, multipotent neural stem cells in the ventricular zone exhibit a bipolar morphology throughout neurogenesis. Progenitors that have acquired a neuronal fate sever their attachments from neural stem cells anchored in the ventricular zone as they move to their designated lamina (Rakic, 2003). During neurogenesis, some of these progenitors, such as intermediate progenitors in the cerebral cortex or granule neuron progenitors (GNPs) of the cerebellum, transition to a multipolar or unpolarized state before terminal differentiation (Singh and Solecki, 2015). In contrast, nonpolarized GNPs of the cerebellar cortex acquire polarity during differentiation, allowing them to undergo directed migration into the internal granular layer (IGL). During this process, the polarity complex must regulate the intercellular adhesions required for migration (Solecki et al., 2004; Famulski et al., 2010). Although cellular polarity transitions appear to occur dynamically throughout neurogenesis, our understanding of cell polarity regulation is limited to signaling cascades and transcriptional networks, which have slow reaction rates. Thus, the main challenge in understanding neurogenesis and neuronal migration is to determine how the dynamic alterations in cellular polarity are regulated. Here, we review the evidence regarding the role of seven in absentia homolog (Siah) ubiquitin ligases in post-translational regulation of polarity and the mechanisms that regulate the interplay between cytoskeletal networks during neuronal migration.

## Siah/Sina

**FIGURE 1:**
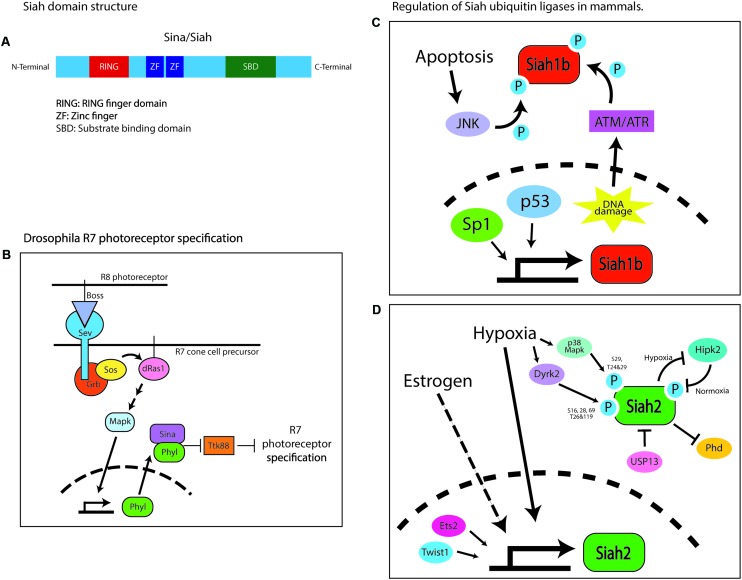
Domain structure of Sina/Siah and its regulation in Drosophila and mammals. **(A)** Domain structure of Siah E3 ubiquitin ligase. **(B)** Schematic of the signaling pathway regulating the specification of the R7 photoreceptor. Sina is placed epistatically downstream of the drosophila Ras–MAPK cascade. Boss, bride of sevenless; Sev, sevenless; Grb, growth factor receptor bound protein; Sos, son of sevenless; Phyl, phyllopod; Ttk88, tramtrack88. **(C)** Schematic of the transcriptional and post-translational pathways involved in regulating Siah1b in mammals. In mice, Siah1b is regulated by p53 while human SIAH1 basal transcription is regulated by Sp1. Post-translationally, Siah1b is regulated through phosphorylation by JNK or ATM/ATM during events of cellular stress like apoptosis and DNA damage. **(D)** Schematic of the transcriptional and post-translational pathways involved in regulating Siah2 in mammals. Estrogen signaling and hypoxia upregulate Siah2 transcriptionally. Twist1 and Ets2 are transcription factors reported to activate transcription of Siah2. Post-transcriptionally, activity of Siah2 is regulated by phosphorylation by multiple kinases – Dryk2, p38, MAPK, and HIPK2, or via physical interaction with USP13. This figure is all original content.

Ubiquitin ligases of the Siah family are composed of the RING catalytic domain, two zinc-finger domains, and a substrate-binding domain (House et al., 2006). The *Drosophila* seven in absentia (SINA) protein was first identified as being required for compound eye development (Carthew and Rubin, 1990). The compound eye consists of 800 identical ommatidia, each composed of clusters of eight photoreceptor neurons, designated R1–R8, surrounded by non-neuronal support cells, including cone cells (Carthew and Rubin, 1990). The photoreceptor lineages are not determined autonomously but through intracellular communication. The specification of each photoreceptor cell depends on the activation of the Ras–MAPK cascade by the *Drosophila* Egf receptor (DER) homolog, except that the R7 photoreceptor specification requires an additional burst of Ras–MAPK activity, which is triggered by another receptor tyrosine kinase, sevenless (sev) (Freeman, 1996). Sev is activated by its ligand bride of sevenless (BOSS), a transmembrane protein expressed by a neighboring R8 cell (Hart et al., 1993). Sev or BOSS loss-of-function (LOF) mutations cause the specific loss of the R7 photoreceptor in each ommatidium (Stark et al., 1976; Reinke and Zipursky, 1988). A recessive viable mutagenesis screen for genes affecting compound eye morphology demonstrated that SINA LOF phenocopies the BOSS or sev mutants. SINA was epistatically placed as the most downstream component of the Ras–MAPK inductive signal, as SINA LOF not only phenocopies loss of the inductive signal but also suppresses ectopic R7 induction driven by active mutants of sev, Ras, or the rolled MAPK (Fortini et al., 1992; Simon, 1994; **Figure 1**). SINA also regulates sensory organ precursor cell fate in a Ras-dependent manner by reducing Notch signaling (Carthew and Rubin, 1990). Subsequent studies have shown that SINA function is regulated by sev and the Ras–MAPK cascade to target for degradation of the *tramtrack* gene product, TTK88, which inhibits R7 photoreceptor specification (Li et al., 1997). SINA-induced TTK88 degradation requires Phyllopod, a Ras-activated adaptor protein that facilitates SINA binding to target proteins (Tang et al., 1997). Finally, suppressor and enhancer screens have revealed SINA regulatory proteins, such as UbcD1, which might be an E2 enzyme for SINA, and the musashi RNA-binding protein that regulates TTK88 translation, along with proteins that synergize with SINA, such as the Sin3a transcription co-repressor (Carthew et al., 1994; Hirota et al., 1999). Thus, *Drosophila* SINA is an adaptable modulator of cell fate that regulates post-translation programs of gene expression via the ubiquitin proteasome system.

In mice, Siah proteins are encoded by three functional genes, designated *Siah1a*, *Siah1b*, and *Siah2*, whereas humans have only two such genes, designated *SIAH1* and *SIAH2* (Della et al., 1993). *Siah1a* and *Siah1b* encode 282-amino acid proteins that are 98% identical, whereas *Siah2* encodes a 325-amino acid protein that is 85% identical to Siah1 but has a longer and highly divergent N-terminus (Della et al., 1993; Hu et al., 1997a). Siah proteins exist as dimers, wherein each monomer consists of an N-terminal RING domain, two zinc-finger motifs, and a C-terminal substrate-binding domain (Polekhina et al., 2002; House et al., 2006; **Figure 1A**). The RING domain and zinc-finger motifs are highly conserved between Sina and Siah (Hu et al., 1997a). Uniquely, the substrate-binding domain of Siah ligases forms a binding groove that specifically recognizes its substrates through a degron motif: Px[ARTE]xVxP (House et al., 2003, 2006). This sequence has remained mostly unchanged during evolution, being nearly identical in flies and vertebrates. Computational screening of the human, mouse, and fly proteomes with the Siah degron also reveals highly conserved targets. With this high degree of conservation of the Siah1 and Siah2 domain structure and degron targeting sequence, it is not surprising that these proteins have overlapping functions in terms of targeting proteins for ubiquitin proteasome degradation (Hu et al., 1997b); however, not only is Siah2 apparently more potent at targeting proteins for degradation, but a small panel of targets can be targeted for degradation only by a vertebrate Siah (Habelhah et al., 2002). Genetic studies demonstrate the functional diversity in *Siah* genes. *Siah1* knockout mice exhibit severe growth retardation, early lethality, and defective meiotic division during spermatogenesis (Dickins et al., 2002), whereas *Siah2* knockout mice exhibit a mild expansion of their myeloid progenitor cells and an altered hypoxia response (a detailed analysis of the brain structure and development has not been carried out in either mutant). The combined loss of *Siah1a* and *Siah2* leads to embryonic and neonatal lethality (Frew et al., 2003). However, this study only provided gross analysis of the brain and lacked any assessment of the cerebellum. Given that *ex vivo* silencing of Siah2 promotes cell cycle and germinal zone (GZ) exit (Famulski et al., 2010), we predict that these mice would exhibit a decrease in proliferation and accelerated differentiation of the GNPs. This will likely lead to reduced number of progenitors and a mild form of cerebellar hypoplasia.

In mammals, Siah ubiquitin ligases play a role in cellular homeostasis and stress response (**Figures 1C,D**). Most notably, in the hypoxic response pathway, Siah targets the prolyl hydroxylases Phd1 and Phd3 for degradation under hypoxic conditions, leading to the stabilization of the transcription factor hypoxia-inducible factor 1a (Hif1a) and the subsequent transcription of key hypoxia-response genes (Safran and Kaelin, 2003; Nakayama et al., 2004). Under normoxic conditions, prolyl hydroxylases target Hif1a for degradation (Bruick and McKnight, 2001; Epstein et al., 2001). Other substrates targeted by Siah are homeodomain-interacting protein kinase (HIPK2) in the DNA damage-response pathway (Calzado et al., 2009); AKAP121 in response to cellular ischemia and oxygen deprivation (Carlucci et al., 2008); GAPDH in hyperglycemia (Yego and Mohr, 2010); TRAF2 in the JNK/p38/NF-κB signaling pathway (Habelhah et al., 2002); TIN2 and Trf2 in cellular senescence (Fujita et al., 2010; Bhanot and Smith, 2012); and Sprouty, the negative regulator of the Ras–MAPK cascade (Nadeau et al., 2007). Siah ubiquitin ligases are also overexpressed in some cancers, including lung cancer (Ahmed et al., 2008), melanoma (Qi et al., 2008), prostate cancer (Qi et al., 2010), breast cancer (Wong et al., 2012), liver cancer (Malz et al., 2012), and pancreatic cancer (Schmidt et al., 2007).

Little is known about how Siah proteins are regulated transcriptionally. p53 regulates *Siah1b* in mice (Fiucci et al., 2004), and Sp1 regulates *SIAH1* basal promoter activity in humans (Maeda et al., 2002). Hypoxia and estrogen signaling have been shown to upregulate Siah2 mRNA (Frasor et al., 2005; Qi et al., 2008). The transcription factors E26 transformation-specific sequence 2, Ets2, and twist-related protein 1, Twist1, cooperate to activate Siah2 transcription in gastric epithelial cells (Das et al., 2016). More is known about how Siah proteins are regulated post-translationally; their abundance and activity are controlled by other interacting proteins or by their phosphorylation status. During hypoxia, Siah2 is phosphorylated by the p38 MAPK at serine 29 and threonines 24 and 29, or by Dyrk2 at serines 16, 28, and 69 and threonines 26 and 119, resulting in enhanced ubiquitination and degradation of the Siah2 substrate Phd3 (Khurana et al., 2006; Pérez et al., 2012). In normoxia, Siah2 is destabilized through phosphorylation at positions 26, 28, and 68 by the HIPK2, which is targeted for degradation by Siah2 during hypoxia (Calzado et al., 2009). A similar regulation pattern is seen with Siah1. For example, Siah1 is stabilized through phosphorylation at tyrosines 100 and 126 by a JNK-dependent pathway during apoptosis or at serine 19 by ATM/ATR in response to DNA damage (Xu et al., 2006; Winter et al., 2008). Besides phosphorylation, Siah2 activity can be modulated by the deubiquitinating enzyme USP13, which binds to and deubiquitinates Siah2, increasing its stability but diminishing its activity toward its substrates (Scortegagna et al., 2011). Hypoxia diminishes USP13 expression, resulting in a Siah2 with increased activity toward its substrates.

## Siah and Neuronal Polarity

The results of recent studies have suggested a function for Siah2 in regulating a complex post-translational network that controls the onset of neuronal polarity during cerebellar granule neuron (CGN) differentiation (Famulski et al., 2010; Trivedi et al., 2017). The CGN is a powerful model with which to understand the linkage between differentiation and polarization in the developing nervous system. During the early stages of postnatal cerebellar development, GNPs proliferate and expand on the surface of the cerebellum. Upon differentiation, the CGNs must migrate radially to their final destinations deep within the cerebellum (Komuro et al., 2001; Leto et al., 2016). To exit the GZ, CGNs depend on the partitioning-defective (Pard) polarity signaling complex to mediate the cell-to-cell attachment and traction forces required for neurons to exit their GZ or migrate. The evolutionarily conserved Pard proteins are archetypal polarity-signaling molecules (Insolera et al., 2011; Nance and Zallen, 2011; McCaffrey and Macara, 2012): Pard3 and Pard6 adaptors form a complex with atypical PKC and CDC42 (Macara, 2004; Suzuki and Ohno, 2006; Goldstein and Macara, 2007) that is crucial for tight-junction formation (Nelson and Beitel, 2009), spindle orientation (Bultje et al., 2009; Sottocornola et al., 2010), cell migration, and axon formation (Barnes et al., 2008). Dissecting Pard protein function has advanced our mechanistic understanding of neuronal migration (Solecki et al., 2004, 2006, 2009; Trivedi et al., 2014; Singh et al., 2016): Both Pard3 and Pard6 are necessary and sufficient for CGN migration. Live-cell imaging revealed that Pard6 is enriched at the centrosome and that CGNs employ a two-stroke nucleokinesis cycle: the centrosome moves forward before somal translocation. Pard6 gain of function (GOF) or LOF disturbed coordinated movement showing that polarity signaling is essential for nucleokinesis (Solecki et al., 2004). Further studies revealed that actomyosin contractions in the CGN leading process generate the forces that propel migration and nucleokinesis. Disrupting Pard6 function halts nucleokinesis by reducing leading-process actomyosin dynamics, force generation, and appropriate leading process extension (Solecki et al., 2009). Moreover, Pard3 activity controls the directionality of CGN motility (Famulski et al., 2010). Polarity is classically defined as asymmetry in cellular architecture, intracellular organization (e.g., epithelial apical-basal polarity, neuronal axon-dendrite polarity) as well as cellular behaviors (e.g., directed migration or intracellular transport). Thus, by controlling events like leading process extension, asymmetric cytoskeletal organization (e.g., directed centrosome motility and actomyosin organization), and the directionality of movement the Pard complex shapes multiples aspects of CGN polarity during their transit to a final laminar position.

**FIGURE 2:**
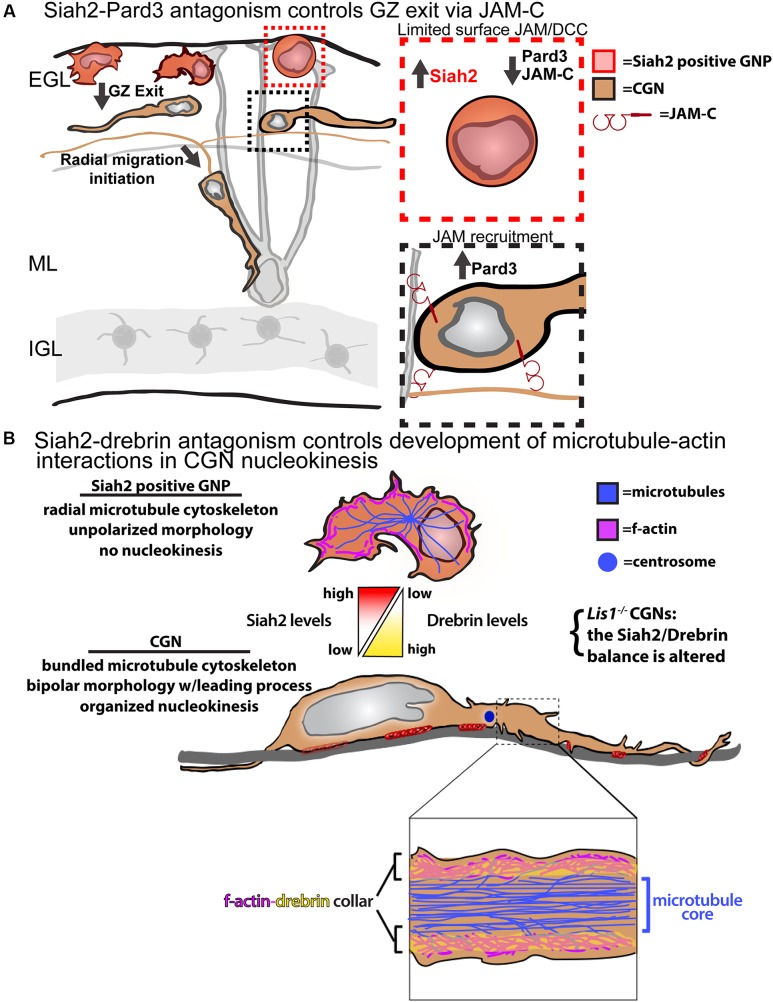
Models for Siah2 antagonism of CGN GZ exit and cytoskeletal interactions. **(A)** Siah2 regulation of Pard3/JAM-C GZ adhesion events. **(B)** Siah2 regulation of microtubule–actin interactions via drebrin. Elements of the **A** panel were derived from Singh et al. (2016). Elements of the **B** panel were derived from Trivedi et al. (2017). Both panels do not require permission for reproduction.

Our laboratory fortuitously discovered that Siah2 regulates polarity signaling in CGN GZ exit and adhesion by controlling Pard-complex activity (**Figure 2A**). Siah2 was the first E3 ubiquitin ligase found to interact with the Pard6 component of the Pard complex via a two-hybrid screen (Famulski et al., 2010). However, a computer-based screen revealed Pard3 to be the only member of the complex harboring a Siah degron sequence, Px[ATRE]xVxP. Siah2 overexpression leads to the ubiquitination and loss of Pard3, but not Pard6, in heterologous cells and primary CGNs, implying that Siah2 regulates polarity via Pard3 degradation. High Siah2 expression in the external germinal layer (EGL) led to the hypothesis that Siah2 regulated CGN GZ exit. *Siah2*^GOF^ blocks CGN migration to the IGL in *ex vivo* slices by restricting motility to the GZ niche, and co-expression of wild-type Pard3 or a degron mutant rescues IGL-directed migration when Siah is elevated. *Siah2* shRNA silencing or expression of a dominant-negative Siah2 spurs GZ exit after 24 h in culture. *In vitro*, *Siah2* overexpression maintained GNPs in a mesenchymal-like progenitor morphology and with a lack of polarized leading process extension while inhibition of Siah2 function produced the opposite phenotypes. Together, these results show that Siah activity inhibits GZ exit partly by antagonizing polarity through the degradation of Pard3. Whereas Pard6 regulates the cytoskeleton required for migration, the Siah2–Pard3 module controls CGN adhesion. JAM-C, a tight-junction molecule localized to the CGN leading process that interacts with Pard3, is also vital for GZ exit. Therefore, Siah2 activity inhibits JAM-C exocytosis and adhesion via Pard3. The mechanism of Siah2 downregulation is unknown, but it enables Pard3 accumulation and the subsequent recruitment of JAM-C to the membrane to initiate GZ exit.

Migrating polarized CGNs undergo a saltatory two-stroke nucleokinesis, in which dilation at the leading edge of the neuron is followed by the inflow of actomyosin complexes and cellular organelles and, finally, by nuclear translocation toward the dilation (Edmondson et al., 1988; Rivas and Hatten, 1995; Solecki et al., 2004, 2009; Tsai and Gleeson, 2005; Trivedi et al., 2014, 2017). The formation of a polarized leading process is fundamental to the polarization of developing CGNs and involves dynamic interplay between f-actin, microtubules, adhesion molecules, and force-generating molecular motors. Microtubule and cytoplasmic dynein plays a role in positioning cellular organelles and towing the nucleus during migration. Actomyosin complexes act within the leading process to mediate the traction forces that arise from the adhesion sites. Our limited understanding of polarized cytoskeletal interplay in migrating neurons, which is mostly investigated using low-resolution imaging, and a lack of candidate molecules to link cytoskeletal elements are key obstacles. Significant recent insights into these processes and how they are connected to neuronal differentiation were made by functionally analyzing the drebrin cytoskeletal adaptor (Trivedi et al., 2017). Drebrin links microtubule movements to the actomyosin cytoskeleton in growth cones and synapses (Geraldo et al., 2008; Geraldo and Gordon-Weeks, 2009; Merriam et al., 2013) but its function in two-stroke motility remains unexplored. Not only is drebrin expression complementary to that of Siah2 in the P7 cerebellum, but *Siah2*^silencing^ led to enhanced drebrin expression in CGNs. Our biochemical studies showed that Siah2 expression reduced drebrin protein levels in a manner requiring Siah2 substrate binding and the VxP degron sequences of drebrin.

Live-cell imaging first indicated that drebrin might be involved in CGN cytoskeletal interplay, as the protein is cyclically transferred from the neuronal soma to the proximal leading process in migration. Lattice light-sheet microscopy, with its superior signal-to-noise ratio and spatial resolution, showed drebrin to be localized to two sub-plasma membrane f-actin-containing collars parallel to the long axis of the CGN leading process that were undetectable at lower resolution. Further examination of the relation of drebrin to plasma membrane, f-actin, and microtubules with super-resolution structured illumination imaging (with approximately 100-nm resolution) showed that the CGN proximal leading process possesses a central core of microtubules and that a layer of drebrin and f-actin intervenes between the plasma membrane and microtubules. These results were surprising, as previous migration models posited that microtubules directly interfaced with the membrane in migrating neurons (Vallee et al., 2009). Functional live-cell imaging studies revealed the drebrin–actin cytoskeletal interface to be critical for CGN migration. Drebrin^silencing^ and dominant-negative inhibition of drebrin–microtubule interactions block the IGL-directed migration of CGNs in *ex vivo* slices without affecting migration speed, inhibit polarized leading-process extension, and randomize centrosome and nucleus movements in nucleokinesis. Finally, epistatic *ex vivo* and *in vitro* live imaging shows that Siah2-drebrin antagonism regulates GZ exit and nucleokinesis. These data imply that actin–microtubule interactions are critical in steering motility and that Siah2-drebrin antagonism is vital for regulating cytoskeletal interactions in developing CGNs (**Figure 2B**). Taken together, these results show that Siah2 regulates CGN migration through several aspects, including cellular adhesion and the cytoskeletal elements that propel migration.

## A Broader Siah2 Polarity Network?

A major goal in developmental biology is to identify polarity-signaling components that will yield insights into the mechanisms controlling tissue morphogenesis. Given the key role of Siah2 in directly regulating cell polarity to control the movement of CGNs to their final laminae, we recently reviewed previous computational screens (Famulski et al., 2010; Trivedi et al., 2017) and noted other Siah2 targets relevant to GZ exit and migration. The same Siah degron screen that implicated Pard3 and drebrin as a Siah target was further analyzed by filtering degron-containing genes via validated CGN expression (via the RIKEN Cerebellar Transcriptome), and pathway analysis revealed common groups of proteins with Siah degrons. Interestingly, Numb, Aspp2 (apoptosis-stimulating of p53 protein 2), and Dab2 (Disabled 2) interact with Pard3 and are also substrates of Siah ubiquitin ligases, suggesting a broader role for Siah2 in polarity regulation. In this section, we will summarize what is known about Siah ligase interactions with these targets in non-neuronal cells and potential implications on the elusive cell biological functions associated with polarization in neurons.

Numb was revealed to be an interacting partner of Siah1 in a yeast two-hybrid screen (Susini et al., 2001). Interaction-mapping experiments and functional analyses confirmed this interaction and showed that endogenous Siah1 targets Numb for degradation in the LTR6 myeloid leukemia cell line. In the developing neocortex, radial glial cells (RGCs) are specialized multipotent progenitors that serve as migratory scaffolds for developing neurons. RGCs are polarized cells with a short apical process that contacts the ventricular surface via the apical end-foot and a long basal process that spans the entire thickness of the developing neuroepithelium (Rakic, 2003). The apical end-feet of RGCs form cadherin-based adherens junctions with each other, anchoring the cells to the ventricular surface (Kadowaki et al., 2007). These cadherin-based adhesions are regulated by Numb and Numb-like (Numbl), homologs of *Drosophila* Numb, an endocytic adaptor protein. In the developing cortex, Numb and Numbl accumulate at the apical end-feet of RGCs, where they co-localize with cadherins, conferring polarity on RGCs (Rašin et al., 2007). Conditional deletion of the *numb* and *numbl* genes affects the cadherin-based adherens junction and disrupts RGC polarity, leading to defects in cortical lamination defects and the generation of neuronal lineages. Given that both Numb and Pard3 have pro-adhesive activities Siah LOF enhancement of RGC junctions could be expected in the developing cerebral cortex of Siah mutant mice. Recently, the tumor suppressor Aspp2 was shown to regulate cell polarity by directly interacting with Pard3 (Sottocornola et al., 2010). This interaction is crucial to recruiting Pard3 to the apical surface of the developing neuroepithelium, the loss of which affects adherens junction formation. Consequently, the loss of Aspp2 affects cortical lamination and interkinetic nuclear migration and strongly perturbs CNS development. Aspp2 is required for tight-junction formation (Kim et al., 2014). Structure–function analysis revealed the presence of a Siah degron motif in Aspp2, the mutation of which abrogated its interaction with Siah2, preventing its proteasome-mediated degradation. Siah2 knockdown and Aspp2 overexpression accelerated tight-junction formation in NRK-52E kidney tubular epithelial cells. During neurogenesis, the maintenance of polarity is crucial to ensure proper cortical lamination, and the loss of polarity at the end of the neurogenic phase is equally important for RGCs to give rise to other non-neuronal cell types. In this context, Siah ubiquitin ligases may mediate the dissolution of adherens junctions by targeting Aspp2 or Numb for degradation, effectively downregulating polarity.

Siah regulation of polarity may extend beyond the adhesion turnover events described above to direct regulation of signaling events. Consistent with this, Pard3 interacts with Dab2, an endocytic adaptor that regulates the endocytosis of certain extracellular receptors and influences the trafficking of cell-signaling components (Traub, 2003). In a recent study, interaction between Pard3 and Dab2 was genetically required to regulate VEGFR endocytosis and turnover in endothelial cells within the angiogenic front in the developing retina (Nakayama et al., 2013). Both Pard3 and Dab2 contain Siah degrons, and Pard3 is a known substrate of Siah2, suggesting that Siah ligases play a role in endocytosis and perhaps in active turnover of Pard complex- and Dab2-linked surface receptors, such as VEGFR or disheveled. Taken together, novel Pard3 interactions in non-neuronal cells coupled with Siah2 regulation suggest a central function of the Pard complex and Siah2 may be related to adhesion and guidance receptor trafficking during neuronal polarization.

## Author Contributions

All authors listed have made a substantial, direct and intellectual contribution to the work, and approved it for publication.

## Conflict of Interest Statement

The authors declare that the research was conducted in the absence of any commercial or financial relationships that could be construed as a potential conflict of interest.
